# Determination of Construction Parameters of Porous Ultra-Thin Overlays Based on Laboratory Compaction Studies

**DOI:** 10.3390/ma13204496

**Published:** 2020-10-10

**Authors:** Jiahao Tian, Sang Luo, Ziming Liu, Xu Yang, Qing Lu

**Affiliations:** 1 School of Transportation, Southeast University, Nanjing 211189, China; 230198289@seu.edu.cn (J.T.); 220173107@seu.edu.cn (Z.L.); 2 Intelligent Transportation Research Center, Southeast University, Nanjing 211189, China; 3 Department of Civil Engineering, Monash University, Clayton, VIC 3800, Australia; xu.yang@monash.edu; 4 Department of Civil and Environmental Engineering, University of South Florida, Tampa, FL 33620, USA; qlu@usf.edu

**Keywords:** small particle size, porous ultra-thin overlay, raveling resistance, construction scheme, energy equivalence principle

## Abstract

To address the severe distresses of asphalt pavement, a new type of pavement maintenance treatment, porous ultra-thin overlay (PUTO) with small particle size was proposed. The PUTO has a thickness of 1.5–2.5 cm and a large void ratio of 18–25%. As a newly asphalt mixture, the structure characteristics differ from poor traditional pavement. Therefore, it is necessary to investigate the fabrication schemes in laboratory and on-site, respectively. In this study, the optimal fabrication schemes, including compaction temperature and number of blows of PUTO were determined based on Cantabro test and volumetric parameters. Then, the corresponding relationship between laboratory and on-site compaction work was then established based on the energy equivalent principle. On this basis, the numbers of on-site rolling passes and the combination method were calculated. The results show that increased compaction temperature and number of blows reduce the height and enhance the compaction of the Marshall sample. With the same temperature and number of blows, the raveling resistance of coarse gradation, Pavement Asphalt Concrete-1 (PAC-1) is better than that of fine gradation, Pavement Asphalt Concrete-2 (PAC-2), and the increased asphalt viscosity significantly improves the raveling resistance of the asphalt mixture. To ensure the scattering resistance and volumetric characteristic, the initial compaction temperature of the PAC-1 and PAC-2 should not be lower than 150 °C and 165 °C, respectively. Then, the laboratory compaction work and on-site compaction work were calculated and converted based on the principle of energy equivalence. Consequently, the on-site compaction combination of rolling machines for four asphalt mixtures was determined. According to the volumetric parameters, the paving test section proved that the construction temperature and the on-site rolling combination determined by laboratory tests are reasonable, and ultra-thin overlay has good structural stability, drainage, and skid resistance.

## 1. Introduction

Under the influence of repetitive traffic loading and changing climatic conditions, surface function distresses of asphalt pavement occur, such as cracking, roughness and insufficient skid resistance, which have significant influences on the comfort and safety of traffic [1]. Therefore, preventive maintenance is prevalent due to the improvements of surface functions and lower initial costs. By conducting preventive maintenance in time, the surface conditions and the original pavement durability can be enhanced [2]. At present, preventive maintenance techniques, including fog seal, slurry seal, micro-surfacing, chip seal, and thin overlay, are used widely [3]. Of these surface treatments, thin overlays are beneficial to improving the defects of roughness and skid resistance. In addition, this surface treatment adopts hot asphalt binder and is more effective in shortening the open time compared with asphalt emulsion treatments, such as micro-surfacing [4].

In this study, a kind of small particle porous ultra-thin overlay (PUTO) is proposed, which has a thickness of 1.5–2.5 cm and a large void ratio of 18–25% [4], and combines the functions of the ultra-thin overlay and the porous drainage asphalt mixture. After the construction of PUTO, interconnected air voids are formed, which play an important role in draining away rainwater and thus improving driving conditions in wet weather. Furthermore, the high air voids in the small particle PUTO is beneficial to reducing traffic noise and improving driving comfort. Therefore, the structural characteristics of the small particle PUTO are prerequisites for achieving satisfactory performance, which are directly related to the construction quality [5]. To ensure the satisfactory formation of the interconnected structure, the construction parameters including compaction equipment, compaction temperature, and compaction scheme, should be determined according to the design parameters.

As a paramount factor in asphalt pavement construction, the compaction process significantly influences the structural formation of asphalt mixtures and the durability of asphalt pavement. During ultra-thin overlay construction, the temperature of the asphalt mixture decreases quickly upon being placed on the original pavement due to the thickness of ultra-thin overlay, which put forwards stricter construction requirements than traditional asphalt mixtures. According to the existing studies, the improper compaction parameters will worsen the performance of pavement in several aspects [6]. To be specific, lower compaction temperature and insufficient compaction energy result in greater air void content, which gives rise to the premature failure of the mixture due to the inferior strength [7]. In contrast, excessive compaction contributes to the loss of drainage function due to the reduction of air voids and failure in the formation of an interconnected structure. Therefore, the construction process and the compaction parameters should be given great importance.

However, few studies have stressed the correlation between laboratory and in-field compaction parameters, as a result, the compaction strategies are generally determined based on construction experience [8]. Currently, the Marshall, Hveem, Bailey, Coupe Aggregate Void Filling (CAVF) and Superpave design methods are the most common design methods for asphalt mixtures. Of these design methods, due to the easy access to test equipment, the Marshall method has been widely used throughout the world since the 1940s [9]. Based on the Marshall design method, Xu [10], Lee [11], and Vackova et al. [12] studied the influences of compaction energy and compaction temperature on specimen performance, including the volumetric properties, the Marshall stability, and the dynamic stability. Furthermore, several studies have been conducted to characterize the correlation of mechanical properties of laboratory specimens and site cores [13,14]. In addition, Micaelo [15], Kassem [16] and Masad et al. [17] analyzed the relationship between different field compaction strategies and laboratory asphalt mixture properties based on digital image processing, field tests and X-ray computed tomography.

Considering the lack of recommended construction scheme in laboratory and on-site for PUTO in the existing specifications, it is necessary to conduct a comprehensive study on both laboratory fabrication and field compaction parameters to supplement the existing specifications. In this study, the laboratory fabrication and on-site compaction scheme were investigated and determined, respectively. Because of the dominant position of the Marshall method in Chinese asphalt pavement design standards and easy access to the experimental equipment, the Marshall method was adopted in this study to fabricate PUTO specimens. The influences of compaction parameters on the raveling resistance of a Marshall sample were analyzed by the Cantabro test. Then the correlation between laboratory and on-site compaction work was established based on the energy equivalent principle. On this basis, on-site compaction schemes were determined and a test section was paved.

The objection of this paper is to determine field compaction parameters of porous ultra-thin overlays proposed in [4], which can be helpful for its construction plan designing. This paper is organized into the following sections. Section 2 shows properties of materials used in this paper and related materials preparation works. The determination of laboratory and on-site compaction schemes is presented in Section 3 and Section 4. Section 5 validate the performance of the paved test section, and Section 6 concludes the study.

## 2. Materials and Preparation

### 2.1. Asphalt

In this research, the styrene-butadiene-styrene (SBS) modified asphalt was used. The typical properties of the modified asphalt binder are shown in Table 1, according to the Standard Test Method of Bitumen and Bituminous Mixture for Highway Engineering in China (JTG E20-2011) [18].

### 2.2. Aggregates

#### 2.2.1. Coarse Aggregate

In this study, a kind of basalt whinstone, Maodi (Yabangkuangye, Changzhou, China) aggregate, was used and its basic properties were tested according to the Testing Procedures of Aggregate for Highway Engineering in China (JTG E42-2005) [19]. The results are shown in Table 2.

#### 2.2.2. Fine Aggregate

Considering the non-negligible effects of fine aggregate properties such as surface roughness and angularity on the Marshall stability and rutting resistance of asphalt mixtures, the typical performance of fine aggregate was tested. The results are shown in Table 3 according to the Testing Procedures of Aggregate for Highway Engineering in China (JTG E42-2005) [19].

### 2.3. Preparation of High-Viscosity Modified Asphalt

**Figure 1:**
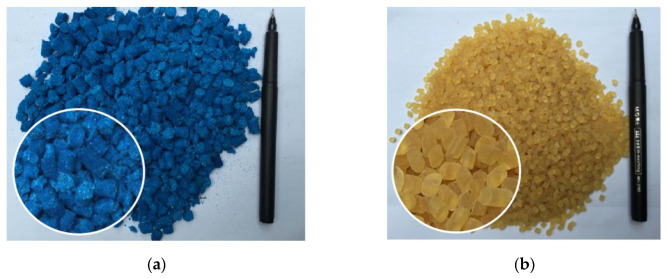
High-viscosity modifiers: (**a**) Type I high-viscosity modifier; (**b**) Type II high-viscosity modifier.

Considering the open-graded aggregate gradation and the point-point contact mode contact mode, the bond strength between asphalt and aggregate is demanding. Therefore, two kinds of high-viscosity modifiers shown in Figure 1, which are commonly used in China, were selected to further modify the SBS-modified asphalt. The basic properties are given in Table 4.

**Figure 2:**
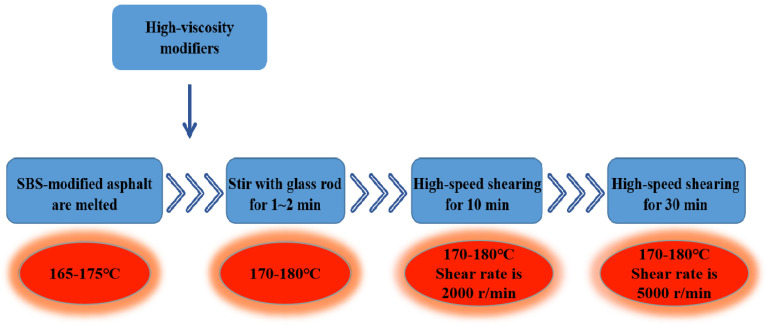
Preparation of high-viscosity modifier-modified asphalt.

A small high-speed shear meter from the PRIMIX company was used to prepare the high-viscosity asphalt binders. The preparation process is shown in Figure 2. After the fabrication of modified asphalt, the dynamic viscosity at 60 °C of the two kinds of modified asphalt were detected according to method T 0620 specified in Standard Test Methods of Bitumen and Bituminous Mixtures for Highway Engineering in China (JTG E20-2011) [18]. According to the results, the dynamic viscosity at 60 °C of the type I and type II modified asphalt are 10.34 × 104 Pa·s and 12.08 × 104 Pa·s, respectively. Therefore, the type II seems more viscous than type I.

### 2.4. Preparation of Asphalt Mixture

**Figure 3:**
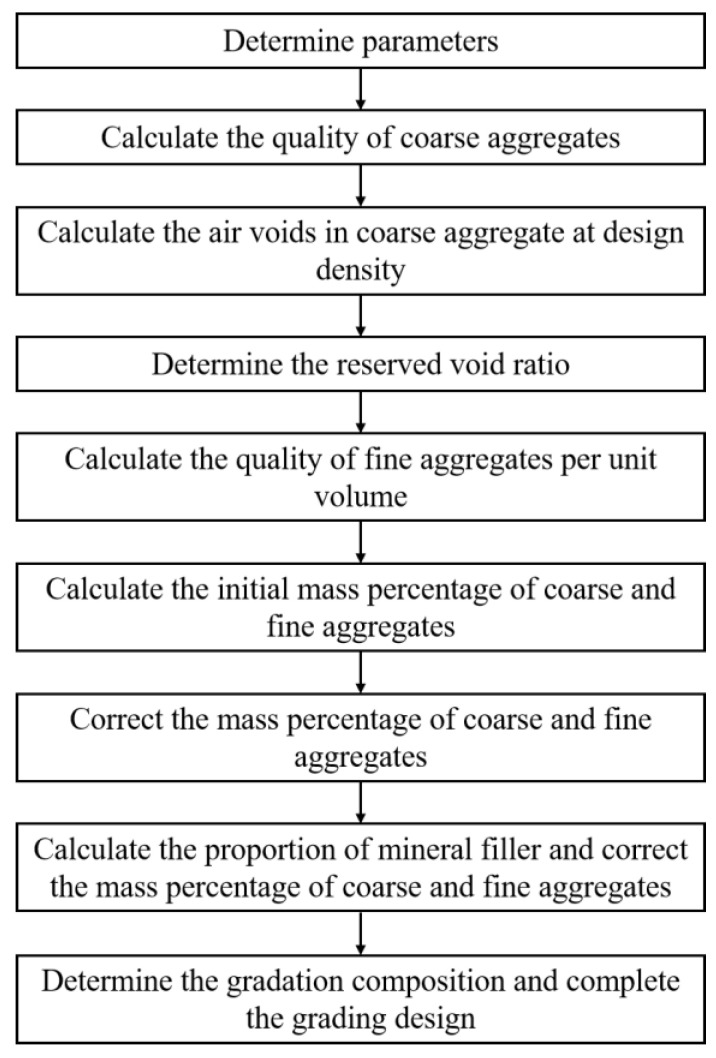
Process of design of aggregate composition.

In this study, the small particle size ultra-thin asphalt mixture was designed according to the flow chart shown in Figure 3. The design of the asphalt mixture mainly involves two phases: determination of aggregate gradation and determination of the optimal asphalt-aggregate ratio.

#### 2.4.1. Determination of Aggregate Gradation

**Figure 4:**
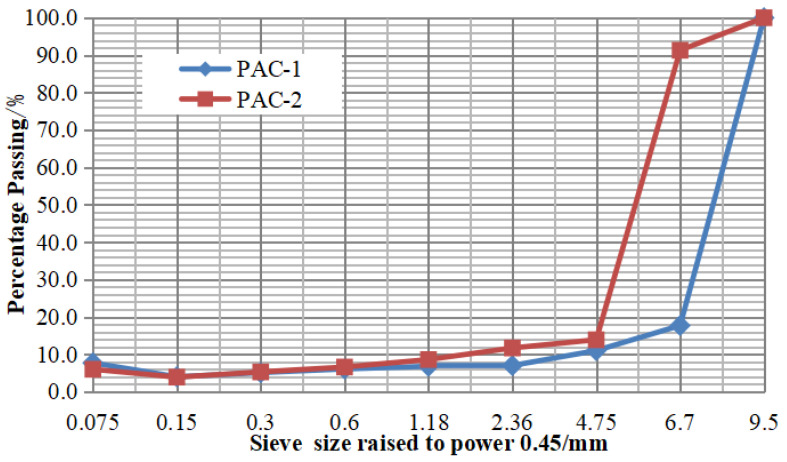
Grading curves of two porous asphalt concretes (PACs).

To evaluate the influence of aggregate gradation on the mechanical properties of the asphalt mixture, two gradations, coarse (PAC-1) and fine (PAC-2), were selected in this study. The nominal maximum particle sizes of PAC-1 and PAC-2 were 9.5 mm and 4.75 mm, respectively. The two designed gradations are shown in Table 5 and the grading curves are shown in Figure 4.

#### 2.4.2. Determination of Optimum Binder Content

Based on the Cantabro test and the Schellenberg Binder Drainage test, the minimum and maximum asphalt contents were determined, respectively. Based on the results, the optimal binder contents for different gradation and modified asphalt combinations were determined and shown in Table 6, where the I suffix in the Mixture type row meaning the type I high-viscosity modifier was adopted and the II suffix represent the type II high-viscosity modifier.

## 3. Determination of Laboratory Compaction Schemes

Laboratory evaluation results of performance of porous ultra-thin overlay can been seen in [20]. In this study, the effects of compaction parameters, including compaction number and temperature, on mixtures mechanical performance were investigated. In particular, the raveling resistances of Marshall samples with different compaction parameters were tested by Cantabro test. In addition, the volumetric parameters of Marshall samples with varying compaction parameters were also measured to determine the optimal compaction schemes in laboratory.

### 3.1. Results of Cantabro Tests

During Marshall samples fabrication process, the number of blows was set for double-sided compaction at 35 times, 50 times, and 65 times. Furthermore, the compaction temperatures were 90 °C, 105 °C, 120 °C, 135 °C, 150 °C, 165 °C and 180 °C, respectively. Then, the raveling resistances of Marshall samples were tested by Cantabro test and the losing weight during the abrasion process was recorded.

#### 3.1.1. Effects of Compaction Number on Raveling Resistance

**Figure 5:**
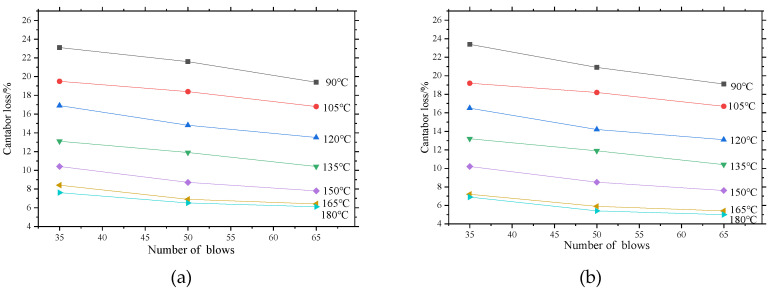

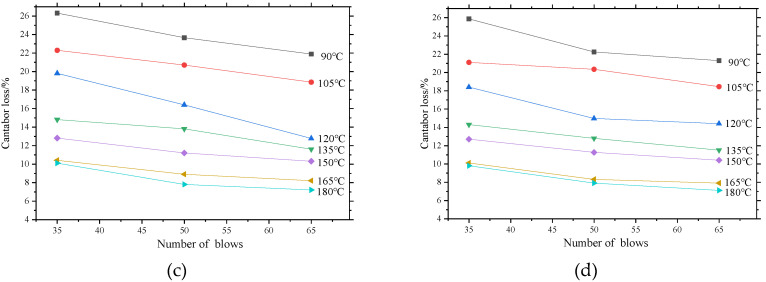
Effects of number of blows on Marshall sample Cantabro loss: (**a**) PAC-1-I, (**b**) PAC-1-II, (**c**) PAC-2-I, (**d**) PAC-2-II.

It can be seen from Figure 5 that under the standard raveling condition (300 revolutions at speed of 30–33 r/min), the Cantabro loss of the four kinds of asphalt mixture decreases with the increase of the number of blows. Moreover, when the number of blows increases from 35 to 50, the decrease in the Cantabro loss of the Marshall sample decreases compared with the increase from 50 times to 65 times. It can be inferred that when the compaction work increases to a certain extent, increasing the number of blows does not have much effect on the improvement of the raving resistance of the mixture. This phenomenon is more pronounced when the compaction temperature reaches 165 °C or higher.

The rate of Cantabro loss of the asphalt mixture with coarse gradation is significantly lower than that of fine gradation with the same compaction scheme. This is speculated that the sample with coarse gradation forms a good skeleton structure during the compaction process. Furthermore, the mutual intrusion between coarse aggregates and the full wrapping of high-viscosity asphalt binder to aggregates make the mixture a stable whole, which effectively diminish aggregate peeling during the raveling process. In contrast, the skeletal structure of fine gradation is weak and thus the mass loss of mixture is significant during the reveling process.

**Figure 6:**
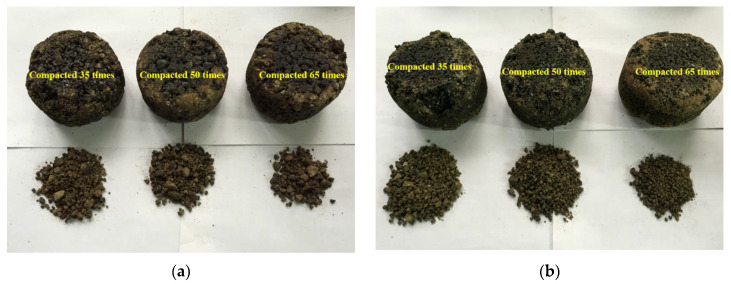
Cantabro loss of Marshall samples with different number of blows (150 °C): (**a**) PAC-1-I; (**b**) PAC-1-II.

The asphalt mixture samples PAC-1-I and PAC-1-II were observed separately, as shown in Figure 6. The figure indicates that the Marshall samples with type II high-viscosity modifier have a lower Cantabro loss rate than samples with the type I high-viscosity modifier. This is mainly related to the viscosity of the two modifier-modified asphalts. The modified asphalt with greater viscosity tends to show better scattering resistance performance. This conclusion is equally applicable to asphalt mixtures PAC-2-I and PAC-2-II.

#### 3.1.2. Effects of Compaction Temperature on Raveing Resistance

**Figure 7:**
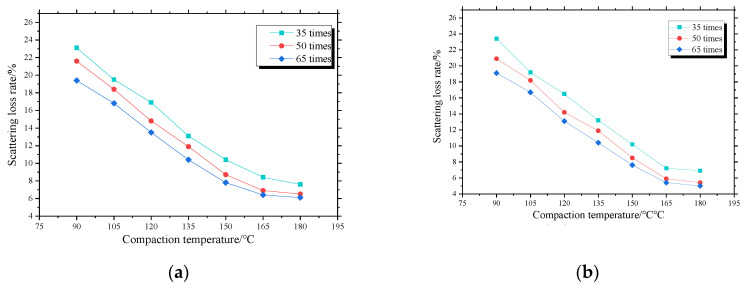

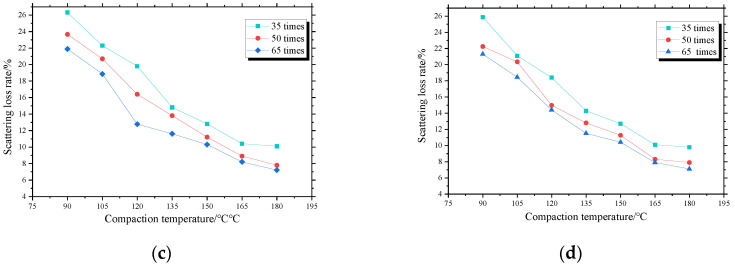
Effects of compaction temperatures on Marshall sample Cantabro loss: (**a**) PAC-1-I, (**b**) PAC-1-II, (**c**) PAC-2-I, (**d**) PAC-2-II.

**Figure 8:**
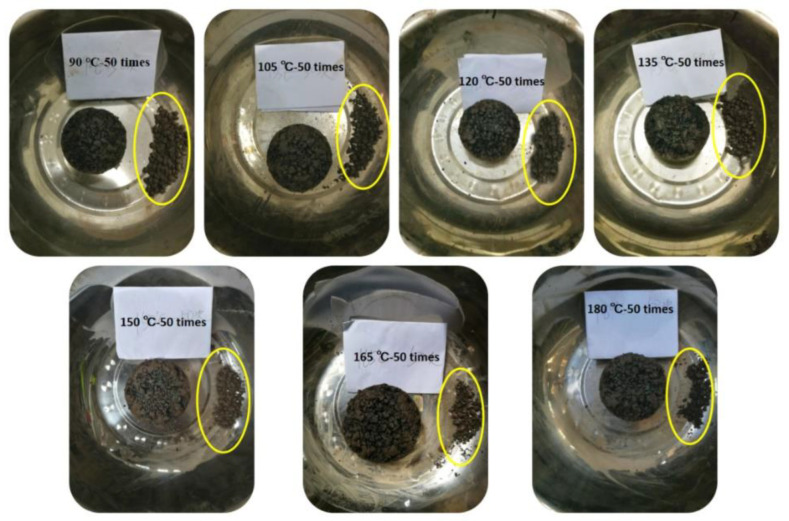
Marshall sample Cantabro loss at different compaction temperatures.

Figure 7 and Figure 8 indicate that for three numbers of blows, the mass loss rates of the four asphalt mixtures decrease with the increase of compaction temperature and decrease becomes smaller and smaller. For asphalt mixtures PAC-1-I and PAC-1-II, when the compaction temperature exceeds 105 °C, the mass loss rates of all samples are less than 20%, meeting the requirements of the specification [21] for open grade asphalt mixtures. When the compaction temperature exceeds 135 °C, the mass loss rates of all samples are less than 15%. When the compaction temperature exceeds 150 °C, the mass loss rates of all samples are less than 10%, with the exception of the sample compacted 35 blows. Therefore, in order to ensure the engineering performance of the overlay, the initial rolling temperature of the asphalt mixture of PAC-1 should not be lower than 150 °C during construction. For the asphalt mixtures PAC-2-I and PAC-2-II, with the exception of the sample that compacted 35 blows. All the samples have a Cantabro loss rate of less than 20%, 15%, and 10%, when the compaction temperature exceeds 120 °C, 135 °C, and 165 °C, respectively. Therefore, the initial rolling temperature of PAC-2 should not be lower than 165 °C in order to ensure the engineering performance of the overlay.

### 3.2. Results of Volumetric Tests

#### 3.2.1. Effects of Compaction Parameters on Samples Heights

**Figure 9:**
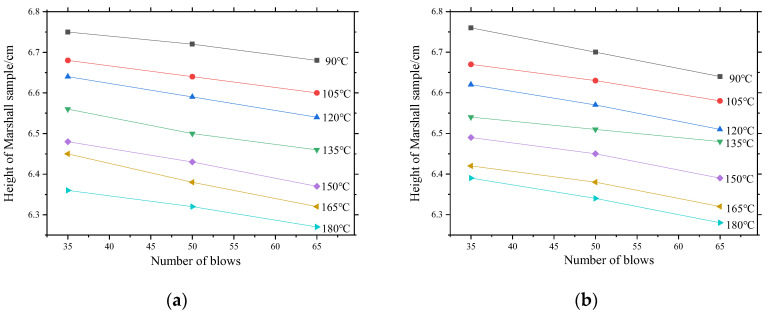

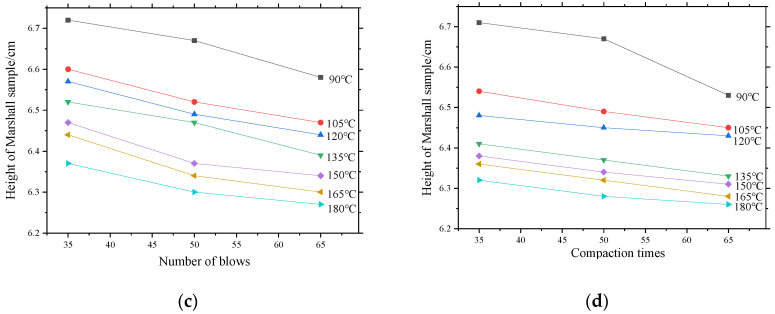
Effects of number of blows on Marshall sample heights: (**a**) PAC-1-I, (**b**) PAC-1-II, (**c**) PAC-2-I, (**d**) PAC-2-II.

The height variation curves of the Marshall samples of the four asphalt mixtures with different numbers of blows are shown in Figure 9. The figure reveals that with different compaction temperatures, the height of the sample decreases as the number of blows increases. For the asphalt mixture PAC-1-I, when the compaction temperature is 150 °C, the height of the Marshall sample with different numbers of blows meets the specification (6.35 ± 0.22 cm). Therefore, 150 °C is considered to be the critical temperature at which the asphalt mixture PAC-1-I reaches an acceptable height. Similarly, the critical temperatures for asphalt mixture compaction for PAC-1-II, PAC-2-I, and PAC-2-II are 150 °C, 150 °C, and 135 °C, respectively.

Furthermore, according to the effects of compaction parameters on raveing resistance, when the compaction temperature is 150 °C and 165 °C in the laboratory, the mass loss rates of the PAC-1 and PAC-2 asphalt mixtures are basically controlled within 10%, and the Marshall samples have excellent raveing resistance. Therefore, the compaction temperatures of PAC-1-I, PAC-1-II were unified as 150 °C and those of PAC-2-I and PAC-2-II were 165 °C, respectively.

#### 3.2.2. Effects of Compaction Parameters on Samples

**Figure 10:**
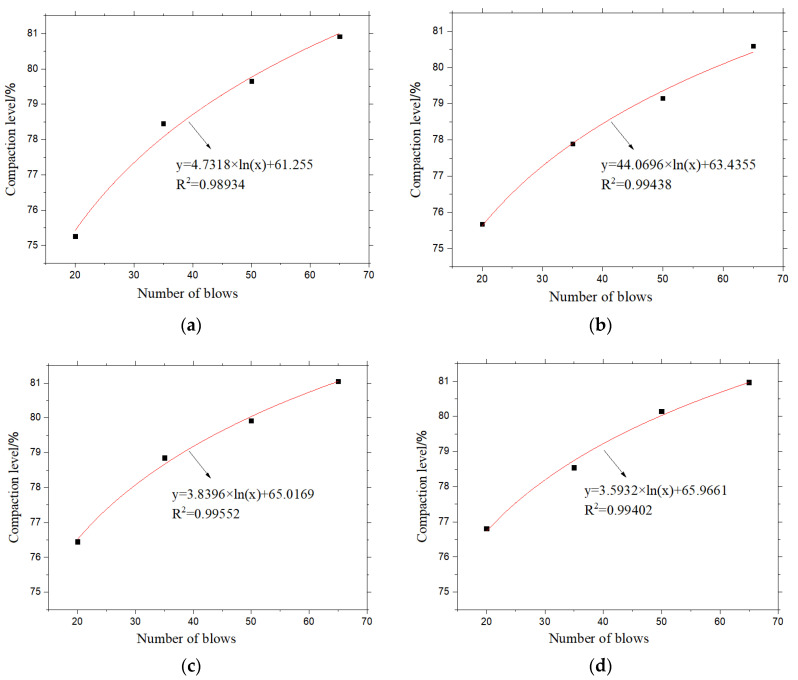
Effects of number of blows on Marshall sample compaction: (**a**) PAC-1-I, (**b**) PAC-1-II, (**c**) PAC-2-I, (**d**) PAC-2-II.

The relationship between the density of the sample and the number of blows is established, and the results are shown in Figure 10. The design void ratio of the four asphalt mixtures are all 20%. It is believed that the target air void is realized when the compaction of the mixture reaches 80%. Therefore, the number of blows when the compaction of the mixture reaches 80% is calculated from the fitting curve, which is the number of blows required for Marshall samples in the laboratory. The number of blows for PAC-1-I, PAC-1-II, PAC-2-I, and PAC-2-II were 53, 59, 50, and 50 times, respectively.

## 4. Correlations of Laboratory and On-site Compaction Schemes

Based on the above studies, the optimal compaction schemes, including number of blows and compaction temperature, of the four kinds of mixtures are determined. To further confirm the on-site compaction schemes, the principle of energy equivalence was used to establish the correlation between laboratory and on-site compaction energy, as shown in Equation (1). In particular, the compaction energy of Marshall samples was calculated and the compaction energy of the construction equipment was determined. Then, the compaction schemes were calculated according to the necessary compaction energy, which enables the mixtures to be compacted with a void ratio of 20%.

(1) $$E_{0}=mgh$$

where *E_0_* is the energy produced by the compaction hammer falling once, J; *m* is the quality of the hammer; *g* is the acceleration of gravity, and 9.8 m/s^2^; *h* is the height at which the hammer is dropped, m.

### 4.1. Calculation of Compaction Energy in Laboratory

A standard Marshall compactor was used to compact the samples in the laboratory. During compaction, a hammer is freely dropped in the vertical direction and continuously compacts the sample to provide the energy required for the Marshall sample to reach a predetermined degree of compaction. The energy calculation method produced by a single compaction is shown in Equation (1). According to the calculations, the energy generated by a single compaction of the hammer is 20.32 J. To achieve a target void ratio of 20%, the number of blows for PAC-1-I, PAC-2-II, PAC-2-I, and PAC-2-II samples were 53, 59, 50 and 50 respectively, and the corresponding compaction works were 1077 J, 1199 J, 1016 J and 1016 J respectively. Obviously, to achieve the same level of compaction, an asphalt mixture with coarse gradation requires more compaction than one with fine gradation. On one hand, the asphalt-aggregate ratio used in the fine gradation is large, and the asphalt film wrapped on the aggregate surface plays a certain lubricating role in the compaction process, which makes the rearrangement between particles easier. On the other hand, due to the larger proportion of coarse aggregates in the coarse gradation, free movement, and mutual intrusion between aggregates with the same volume are more difficult, increasing internal frictional resistance.

### 4.2. Calculation of Compaction Energy on Site

The compaction of asphalt pavement is generally divided into three stages: preliminary compaction, re-compaction, and final compaction. Since the purpose of preliminary compaction is to make the loose mixture relatively stable, small-tonnage compaction machines are mainly used. Re-compaction is a key process in compaction and plays a major role in the compaction of the asphalt mixture. Static compaction or vibratory compaction is generally selected, as needed. The final compaction is to eliminate previous rolling traces and further stabilize the aggregate. Generally, a rubber or steel roller is used to roll the surface pavement.

#### 4.2.1. Compaction Energy Generated by Paver

Before being compacted by a steel roller or rubber roller, the loose asphalt mixture is pre-compacted by the screed at the rear of the paver. It also plays a significant role in the process of mixture compaction. A study [22] indicates that the energy provided by the paver screed to the asphalt mixture is equivalent to the energy produced by a Marshall sample being compacted 15 blows in the laboratory. Therefore, the pre-compaction energy provided by the paver for the mixture is calculated and shown in Equation (2).

(2) $$E_{p}=nE_{0}=15\times 20.32=304.8\;J$$

where *E_p_* is the energy of paver pre-compacting, J; and *n* is the number of blows.

#### 4.2.2. Compaction Energy Generated by Steel Roller

The steel roller generally has the dual functions of static and vibration compaction. Static compaction relies on the static pressure generated by its own weight to cause the relative movement of the aggregate. Vibratory compaction causes the material to resonate with the steel wheel through vibration impact force, and the exciting force propagates further in the depth direction, so that a thick mixture can also obtain a better compaction effect. However, a large vertical exciting force may cause coarse aggregates on the surface of the pavement to be crushed, and “white spots” on the pavement surface appear. To avoid this phenomenon, and taking into account the thin structural features of the overlay, vibration compaction is not used in the compaction process. The energy absorbed by asphalt mixture for one pass (back and forth) can be obtained by changing the height of the asphalt pavement, as shown in Equation (3):

(3) $$E_{s}=F_{s}h_{s}$$

where *E_s_* is the energy provided by the steel roller for 1 pass, J; *F_s_* is the weight of the steel roller, N; and *h_s_* is the pavement thickness variation with the steel roller rolling once, m.

According to the measured density change of the mixture during construction process, the height change for one pass of the asphalt mixture can be calculated. After the mixture was pre-compacted by the paver, it was then compacted for 4 blows by a steel roller, and the height change (Δ*h*) of the pavement was 0.2 cm, and the average height change (*h_s_*) per compaction was 0.05 cm [23].

#### 4.2.3. Compaction Energy Generated by Rubber Roller

Due to the elasticity of the rubber pneumatic tire, a rubber roller applies horizontal and vertical forces to the pavement, thereby causing a smashing effect on the asphalt mixture. However, since the horizontal force does not directly affect the enhancement of the pavement density, only vertical force is considered in the calculation. Similar to the static pressure of a steel roller, the rubber roller also compacts the mixture by gravity. The work done on the asphalt mixture for one pass is *Er*, and can be obtained by Equation (4):

(4) $$E_{r}=F_{r}h_{r}$$

where *E_r_* is the energy provided by the rubber roller for 1 pass, J; *F_r_* is the weight of rubber roller, N; *h_r_* is the pavement thickness variation of steel roller rolling once, m.

After the rolling of the rubber roller, it can also be calculated that the average height change of pavement compacted by the rubber roller is about 0.04 cm (*h_r_* = 0.04 cm), based on the measured density change of mixture at the construction site [22].

### 4.3. Determination of Compaction Schemes on Site

Based on the principle of energy equivalence, it is known that an asphalt mixture requires energy to achieve a predetermined degree of compaction, and the energy is absorbed by the asphalt mixture from the paver, steel roller and rubber roller, respectively. Therefore, the number of rolling passes of the steel or rubber roller is determined. A DD130 steel roller and XP301 rubber roller were used in the paving process of this test section, and their masses are 13.442 t and 30 t respectively. According to the above equation, the energy provided by rolling one pass with the steel roller and rubber roller is 65.9 J and 117.6 J, respectively. Taking the PAC-1-I asphalt mixture as an example, the required compaction work is 1077 J when the target void ratio is 20%, of which 304.8 J is provided by the paver, and the remaining 772.2 J is provided by the compaction machine. Therefore, the number of rolling passes of the steel and rubber roller can be calculated by Equation (5):

(5) $$E=E_{p}+n_{s}E_{s}+n_{r}E_{r}$$

where *E* is the energy required for the asphalt mixture to reach a predetermined density, J; *n_s_* is the number of rolling passes of the steel roller; and *n_r_* is the number of rolling passes of the rubber roller.

The on-site specific rolling number was calculated according to Equation (5), and the two best calculated compaction methods for each asphalt mixture were selected. As shown in Table 7, the calculated number of passes for the PAC-1-I asphalt mixture is 3 and 5 or 5 and 4, respectively (*n_s_* = 3, *n_r_* = 5 or *n_s_* = 5, *n_r_* = 4). The calculated number of passes of the PAC-1-II asphalt mixture is 3 and 6 or 5 and 5, respectively (*n_s_* = 3, *n_r_* = 6 or *n_s_* = 5, *n_r_* = 5). The number of rolling passes for the PAC-2-I and PAC-2-II asphalt mixtures are 2 and 5 or 4 and 4, respectively (*n_s_* = 2, *n_r_* = 5 or *n_s_* = 4, *n_r_* = 4). The final on-site specific number of passes for the steel roller and rubber roller can be rationally determined according to engineering experience and the compaction machinery inventory.

## 5. Test Section Paving

### 5.1. Introduction of the Test Section

**Figure 11:**
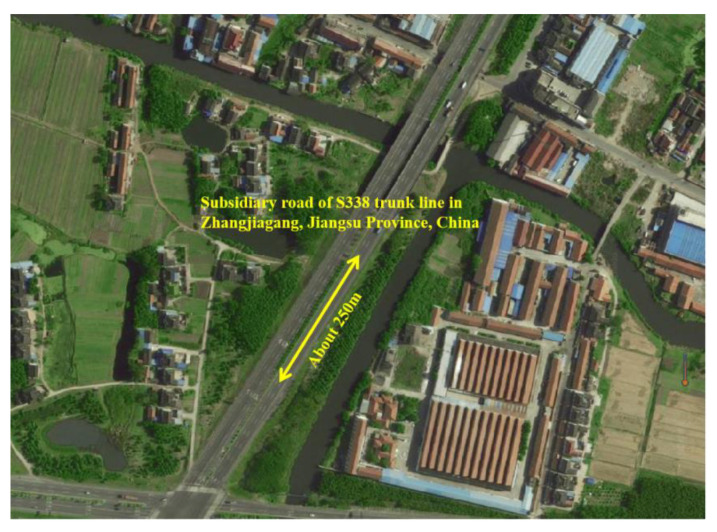
Position of test section.

The location of the test section is near a subsidiary road on the S338 trunk line in Zhangjiagang, Jiangsu Province, China. The test section has a total length of about 250 m and a width of 11.75 m, as shown in Figure 11. The PAC-1-II asphalt mixture was used for paving. The thickness of the overlay is 2.0 cm, the tack coat is made of SBS-modified emulsified asphalt, and the sprinkling rate is controlled at 0.8–1.0 L/m^2^. Because the lower layer is a new pavement of SMA-16, there was no need for special treatment of the lower layer before construction. The test section was paved in September 2018. The overall construction quality was excellent, the surface of pavement was flat and compact, and there were no obvious rolling traces.

### 5.2. Construction Process

#### 5.2.1. Spread of Tack Coat

**Figure 12:**
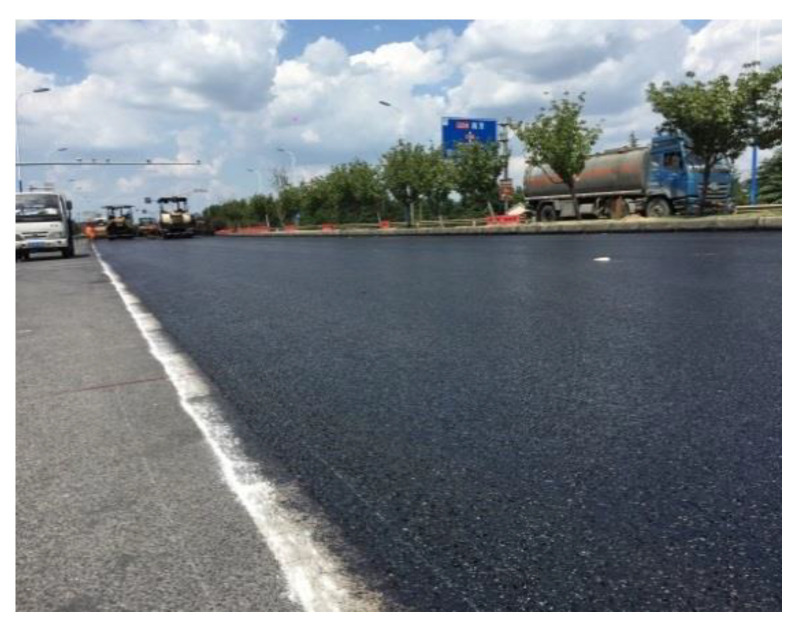
Scene after construction of tack coat.

In rainy weather, in order to prevent rainwater from infiltrating from the ultra-thin overlay, entering the lower layer and causing water damage to the pavement structure, it is necessary to spread a tack coat between the overlay and the lower layer. This not only prevents rainwater from further infiltrating the pavement, but also facilitates the lateral discharge of accumulated water. SBS-modified emulsified asphalt was used in the test section, the spraying rate was controlled at 0.8–1.0 L/m^2^, and sprinkling work was completed within 4–6 h before the construction of the overlay. Figure 12 shows the scene after construction of the tack coat.

#### 5.2.2. Plant Mixing

**Figure 13:**
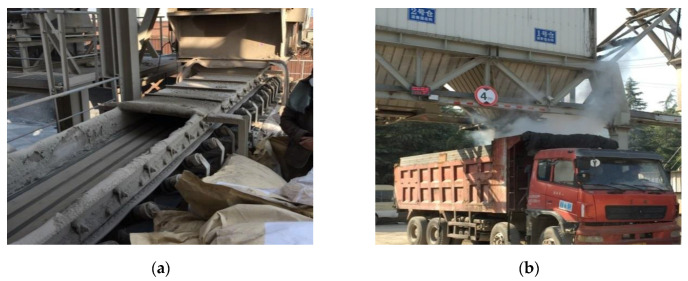
Feeding and charging process: (**a**) High-viscosity modifier feed port; (**b**) A truck being loaded.

According to the production capacity of the mixing plant, the proportion of aggregate, mineral powder, the quality of SBS-modified asphalt and high-viscosity modifier of each mixing was 2.5 t. The mixing temperature of the asphalt mixture must be strictly controlled. Asphalt mixture with a mixture temperature lower than the lower limit of 170 °C or higher than the upper limit of 195 °C must be discarded, and the mixing temperature is preferably between 180–185 °C. When there was sufficient mix in the warehouse, the warehouse was opened and discharged, and a total of five trucks transported the asphalt. The loading of each truck was controlled at 20–30 t. The feeding and charging process are shown in Figure 13.

#### 5.2.3. Asphalt Mixture Paving

**Figure 14:**
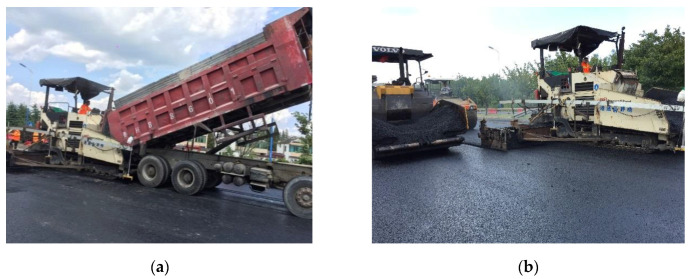
Paving process: (**a**) asphalt mixture feeding; (**b**) placement and paving.

Firstly, the asphalt mixture temperature was measured, as the specified temperature should not be lower than 165 °C. The unloading trucks need to be insulated to prevent the temperature from falling too fast. Two asphalt mixture pavers were used for joint paving, which were 5 to 10 m apart. Each paver is equipped with a temperature display device to measure the temperature of the asphalt mixture during the paving process. The paving temperature was controlled at between 155 and 170 °C, and the thickness of loose paving was between 2.2 and 2.3 cm. The unloading and paving process is shown in Figure 14.

#### 5.2.4. Asphalt Mixture Compaction

**Figure 15:**
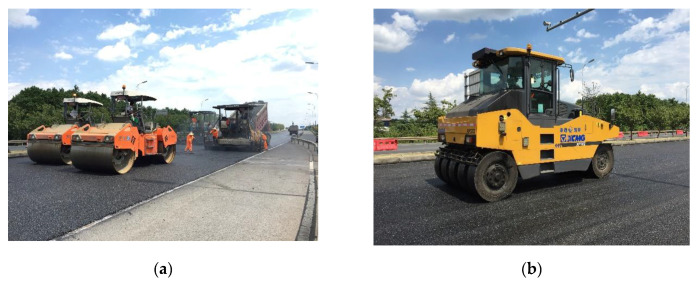
Compaction process: (**a**) Compaction by steel roller; (**b**) Compaction by rubber roller.

The compaction work was carried out immediately after the asphalt mixture had been paved. For the new and old working face joints, a small vibratory compactor was used to repeatedly compact 5 to 6 times. After the joint was processed, the steel roller started to compact. Please note that in order to prevent sticking and prevent the temperature of the asphalt mixture dropping too fast, the steel roller and rubber roller were manually oiled, and no watering was carried out. According to the results reported in above sections, the initial compaction temperature was set to 150 to 165 °C, and the steel roller was used to compact the pavement 2 times. The intermediate was carried out immediately after breakdown by using steel roller to compact the pavement 3 times. Finally, the rubber roller was used to compact the pavement 5 times. The compaction process is shown in Figure 15.

### 5.3. Field Survey of Test Section

#### 5.3.1. Void Ratio

**Figure 16:**
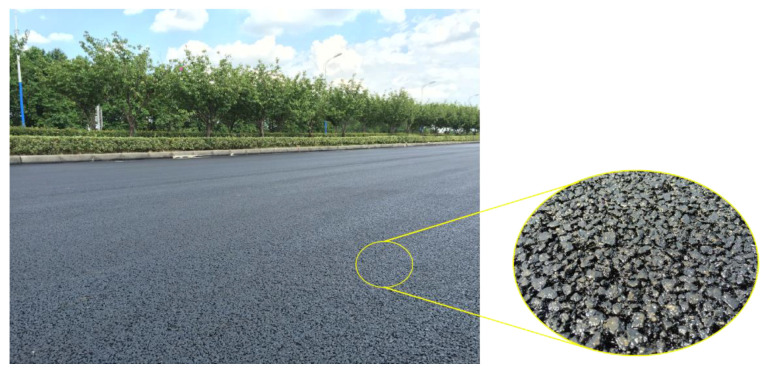
Paving completed.

**Figure 17:**
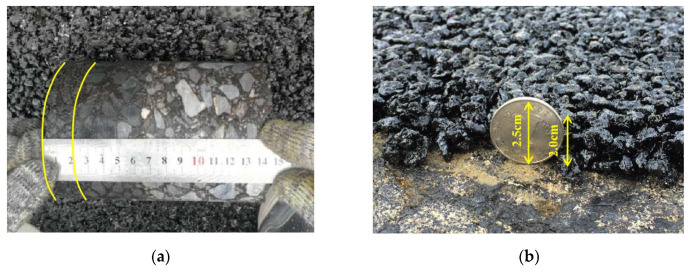
Verification of thickness of ultra-thin overlay: (**a**) Measuring core sample drilled at site; (**b**) Roadside measurement.

**Figure 18:**
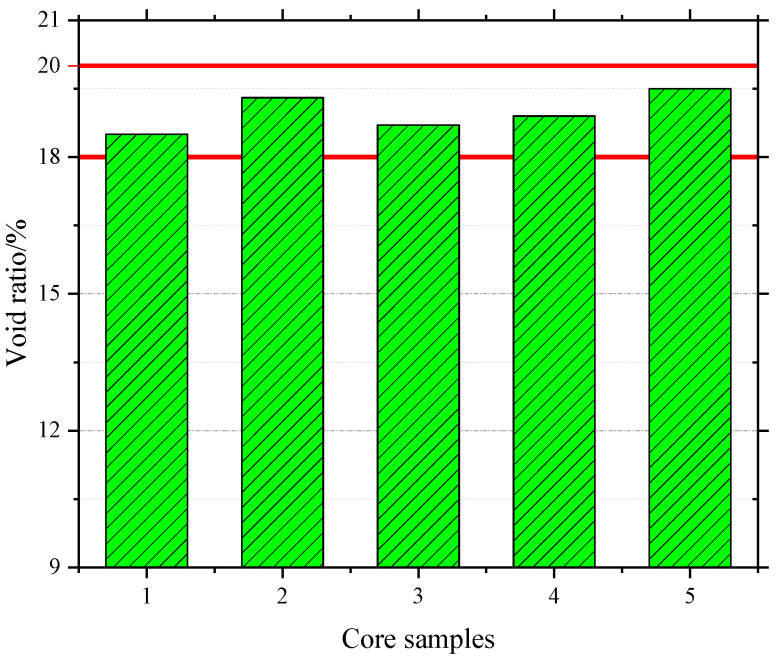
Void ratio of core samples.

After the construction, as shown in Figure 16, the surface of the test section had good flatness and density, and the overall quality of construction was excellent. On the second day after the construction, a core sample was taken to measure the thickness of the overlay, as shown in Figure 17. The thickness of the overlay was 2.0 cm, which meets the design requirements. In addition, five core samples were taken to test the air void content. The results shown in Figure 18 are within the target air void content range of 18–20%. Therefore, the structural performance is assured. Finally, some test measures were taken to evaluate the drainage and skid resistance properties of the overlay.

#### 5.3.2. Drainability

**Figure 19:**
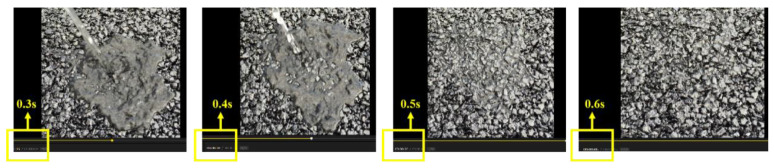
Drainage test.

**Figure 20:**
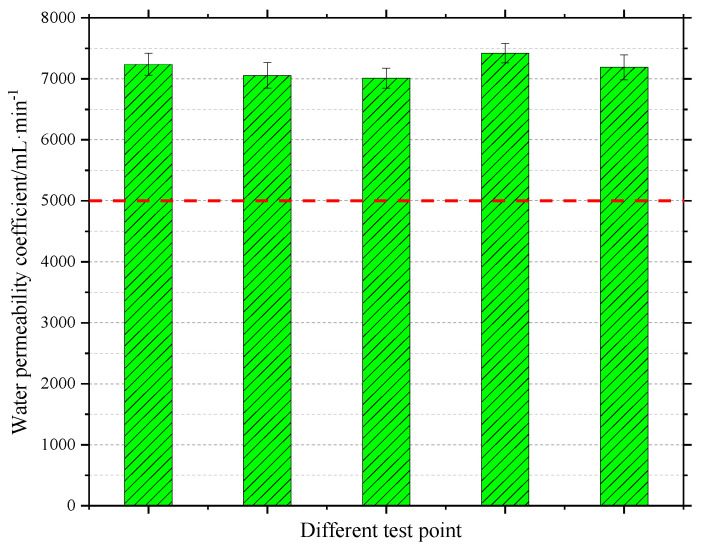
Water permeability coefficients at different test points.

Pouring water onto the pavement was used to simply determine the drainage performance of the overlay. A video screenshot is shown in Figure 19. It can be clearly seen that the overlay drains quickly, and it takes less than 3 s from the start of pouring water to drain all the water. It appears that the overlay has rich interconnected voids inside it to meet the drainage requirements. To verify the drainage performance of the overlay more accurately, a drainage coefficient test was carried out to evaluate drainage performance according to the T 0623 method specified in Standard Test Methods of Bitumen and Bituminous Mixtures for Highway Engineering in China (JTG E20-2011) [18]. The test results shown in Figure 20 indicate that the water drainage coefficient of the overlay at different measuring points reached 7000 mL/min, indicating that the drainage performance of the test section is excellent.

#### 5.3.3. Skid Resistance

**Figure 21:**
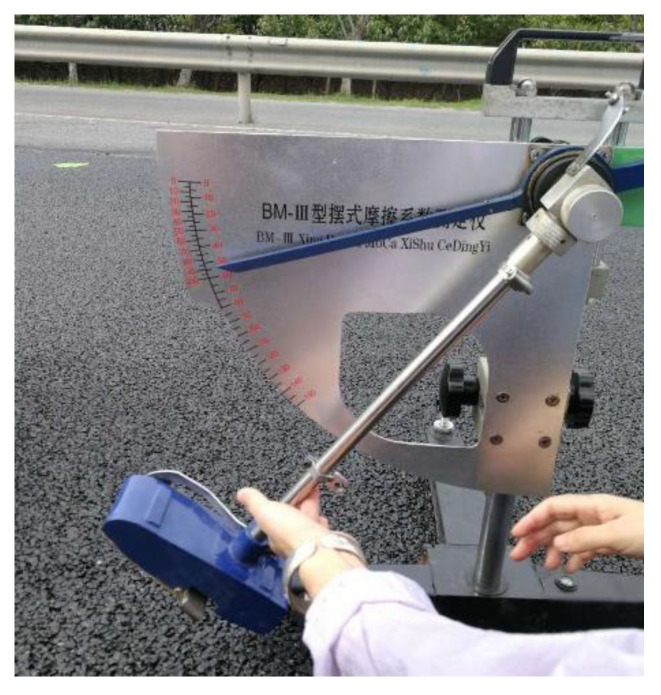
Pendulum friction test.

**Figure 22:**
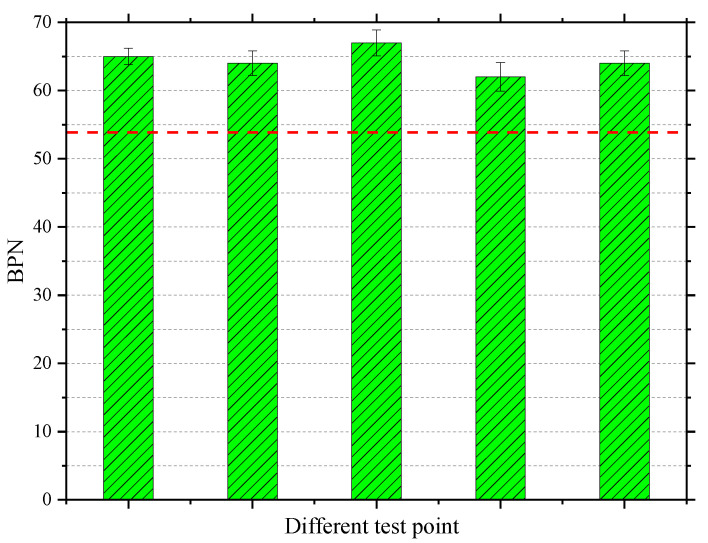
BPNs at different test points.

The pendulum friction test and sanding patch method was used to evaluate the skid resistance of the test section, as shown in Figure 21. Figure 22 shows that the British Pendulum Number (BPN) value obtained by the pendulum friction test is about 64, which satisfies the specification requirements [21] and ensures the skid resistance of vehicles during low-speed travel. In addition, continuous road performance surveys have been conducted since the PUTO construction with an interval of 12 months and the BPN value of the original pavement was also detected, as shown in Table 8. According to the survey results, although the BPN value of PUTO represent slight downwards trend, the BPN values of PUTO are always greater than that of original pavement, even after 2 years. Therefore, the PUTO does show great skid resistance.

## 6. Conclusions

In this study, the influences of compaction temperature and number of blows on the PUTO Marshall samples raveling resistance were analyzed by Cantabro test. Consequently, the optimal fabrication schemes of PUTO were determined. Then, the corresponding relationship between laboratory and on-site compaction work was then established based on the energy equivalent principle. On this basis, the numbers of on-site rolling passes and the combination method were calculated. Finally, a test section was paved, and the performance of the test section was verified after paving. Based on the results and discussion, the following conclusions can be drawn:

(1) Increased number of blows and compaction temperature effectively reduces the height of Marshall samples and enhance the density of samples.
(2) For the same number of blows and compaction temperature, the mass loss rate of coarse gradation (PAC-1) is lower than that of fine gradation (PAC-2), and increasing the asphalt viscosity significantly improves the raveling resistance of the asphalt mixture.
(3) To meet the requirement that the Cantabro loss rate of the Marshall sample is less than 10%, the initial compaction temperature of the PAC-1 asphalt mixture should not be lower than 150 °C, and the initial compaction temperature of the PAC-2 asphalt mixture should not be lower than 165 °C during construction.
(4) Based on the principle of energy equivalence, the correlation between laboratory and on-site compaction work was established and consequently the on-site compaction combination of rolling machines for four asphalt mixtures was determined.
(5) According to the volumetric parameters, the paving test section proved that the construction temperature and on-site rolling combination determined by laboratory tests are reasonable; Furthermore, the ultra-thin overlay has good structural stability, drainage, and skid resistance.

## Figures and Tables

**Table 1:** Properties of SBS modified asphalt.

Test Items		Test Results	Standard Results	Test Methods
Penetration (25 °C 100 g, 5 s)/0.1 mm		68.8	60–80	T 0604-2011
Softening point (TR&B)/°C		64.6	>55 °C	T 0606-2011
Ductility (5 °C 5 cm/min)/cm		52.8	≥30	T 0605-2011
Penetration index		0.26	≥−0.4	T 0604-2011
Flexible recovery (25 °C)/%		79	≥65	T 0662-2011
Density (25 °C)/g/cm^3^		1.079	N/A	T 0603-2011
After TFOT	Quality loss/%	−0.01	≤±1.0	T 0609-2011
	Penetration ratio (25 °C)/%	73	≥60	T 0604-2011
	Ductility (5 °C)/cm	28	≥20	T 0605-2011

**Table 2:** Properties of coarse aggregate.

Test Items	Test Results	Standard Results	Test Methods
Soft stone content/%	0.6	≤1.0	T0320-2005
Crushed ratio/%	10.5	≤18	T0316-2005
Apparent density/g/cm^3^	2.991	≥2.70	T0304-2005
Water absorption rate/%	0.8	≤1.0	T0304-2005
Los Angeles wear loss/%	15	≤20	T0317-2005
Rugged/%	3.5	≤8	T0314-2005
Needle and flake stone content/%	1.4	≤12	T0312-2005

**Table 3:** Properties of fine aggregate.

Test Items		Test Results	Standard Results	Test Methods
Rugged/%		2.1	≤3	T0340-2005
Apparent relative density/g/cm^3^	1.18–4.75 mm	2.952	≥2.50	T0328-2005
	0.15–2.36 mm	2.927		
Sand equivalent/%		71.3	≥60	T0334-2005
Methylene blue value/g/kg		0.8	≤1.5	T0349-2005
Crushed value		6	≤10	T0350-2005
Angularity/%		33	≥30	T0345-2005
clay content/%		1.8	≤3.0	T0333-2005

**Table 4:** Properties of high-viscosity modifiers.

Test Items	Test Results		Standard Results	Test Methods
	Type I High-Viscosity Modifier	Type II High-Viscosity Modifier		
Exterior	Blue particles, evenly dispersed	Yellow particles, evenly dispersed	Granular, evenly dispersed	Visual inspection
Individual particle quality/g	0.14	0.05	≤0.5	Weighing
Ash/%	0.35	0.4	≤1.0	T 0614-2011
Dry mix dispersibility	A little residue	No residue	No residue	Dry mix

**Table 5:** Gradation of two porous asphalt concretes (PACs).

Gradation Type	Mass Percentage of Aggregate through Each Sieve/%							
	9.5 mm	4.75 mm	2.36 mm	1.18 mm	0.6 mm	0.3 mm	0.15 mm	0.075 mm
PAC-1	100.0	17.7	11.0	7.0	6.8	6.1	5.1	4.0
PAC-2	100.0	91.3	13.9	11.7	8.6	6.6	5.3	3.9

**Table 6:** Optimal binder content.

Mixture Type	PAC-1-I	PAC-1-II	PAC-2-I	PAC-2-II
Optimal binder content	4.4%	4.3%	5.0%	5.0%

**Table 7:** Compaction method for four asphalt mixtures.

Mixture Type	E_0_(J)	Compaction Method1			Compaction Method2		
		*n_s_*	*n_r_*	E(J)	*n_s_*	*n_r_*	E(J)
PAC-1-I	1077	3	5	1090.5	5	4	1104.7
PAC-2-I	1199	3	6	1208.1	5	5	1222.3
PAC-1-II	1016	2	5	1024.6	4	4	1038.8
PAC-2-II	1016	2	5	1024.6	4	4	1038.8

**Table 8:** The BPN value of the PUTO and original pavement.

Duration after Construction	0 Month	12 Months	24 Months
BPN of PUTO	64	62	61
BPN of original pavement	58	57	57

## References

[1] Zhou Q.F. (2018). Research on Performance of Skid Resistance and Noise Reduction of Ultra-thin Bonding Protective Layer of Asphalt Pavement.

[2] Dept. of Transportation, Federal Highway Administration (2000). Insights into Pavement Preservation—A Compendium.

[3] Zhang H., Li H., Zhang Y., Wang D., Harvey J., Wang H. (2018). Performance enhancement of porous asphalt pavement using red mud as alternative filler. Constr. Build. Mater..

[4] Liu Z., Wang X., Li Q., Yang X., Li Q. (2019). Asphalt mixture design for porous ultra-thin overlay. Constr. Build. Mater..

[5] Airey G., Collop A. (2014). Mechanical and structural assessment of laboratory- and field-compacted asphalt mixtures. Int. J. Pavement Eng..

[6] Hunter A.E., McGreavy L., Airey G. (2009). Effect of Compaction Mode on the Mechanical Performance and Variability of Asphalt Mixtures. J. Transp. Eng..

[7] Suresha S., Varghese G., Shankar A.R. (2009). Characterization of porous friction course mixes for different Marshall compaction efforts. Constr. Build. Mater..

[8] Hayat A., Hussain A., Afridi H.F. (2019). Determination of in-field temperature variations in fresh HMA and corresponding compaction temperatures. Constr. Build. Mater..

[9] Kuanghuai W., Xiaoning Z. (2005). Summarization on methods of asphalt mixture design. J. Guangzhou Univ. Nat. Sci. Ed..

[10] Xu B., Chen J., Zhou C., Wang W. (2016). Study on Marshall Design parameters of porous asphalt mixture using limestone as coarse aggregate. Constr. Build. Mater..

[11] Lee S.-J., Amirkhanian S.N., Kwon S.-Z. (2008). The effects of compaction temperature on CRM mixtures made with the SGC and the Marshall compactor. Constr. Build. Mater..

[12] Vacková P., Valentin J., Kotoušová A. (2017). Impact of lowered laboratory compaction rate on strength properties of asphalt mixtures. Innov. Infrastruct. Solut..

[13] Mitchell M.R., Link R.E., Wang W., Höeg K. (2009). Method of Compaction has Significant Effects on Stress-Strain Behavior of Hydraulic Asphalt Concrete. J. Test. Eval..

[14] Yu J.M., Li Z., Zhang X.N. (2011). Validity Evaluation of Different Laboratory Asphalt Concrete Material Compaction Methods with Digital Image Processing Technique. Adv. Mater. Res..

[15] Micaelo R., Azevedo M.C., Ribeiro J. (2014). Hot-mix asphalt compaction evaluation with field tests. Balt. J. Road Bridg. Eng..

[16] Kassem E., Masad E., Chowdhury A., Claros G. (2008). Influence of field compaction pattern on asphalt pavement uniformity. J. Assoc. Asph. Paving Technol..

[17] Masad E., Scarpas A., Rajagopal K.R., Kassem E., Koneru S., Kasbergen C. (2016). Finite element modelling of field compaction of hot mix asphalt. Part II Appl. Int. J. Pavement Eng..

[18] General Administration of Quality Supervision, Inspection and Quarantine of the People’s Republic of China (2011). Standard Test Method of Bitumen and Bituminous Mixture for Highway Engineering in China.

[19] General Administration of Quality Supervision, Inspection and Quarantine of the People’s Republic of China (2005). Testing Procedures of Aggregate for Highway Engineering in China.

[20] Liu Z., Li Q., Quan X., Wei X., Yang X., Li Q. (2019). Laboratory evaluation of performance of porous ultra-thin overlay. Constr. Build. Mater..

[21] General Administration of Quality Supervision, Inspection and Quarantine of the People’s Republic of China (2004). Technique Specification for Construction of Highway Asphalt Pavement in China.

[22] Hanguang L., Ying G., Bin Y.W. (2011). Compaction characteristics of asphalt mixture and determination of rolling pass number of asphalt pavement. J. Southeast Univ. Nat. Sci. Ed..

[23] Zhenhe H. (2012). Study on Reasonable Compaction Method and Mechanical Configuration of New Materials for Asphalt Pavement.

